# Is there a “pandemic effect” on individuals’ willingness to take genetic tests?

**DOI:** 10.1038/s41431-022-01223-6

**Published:** 2022-11-10

**Authors:** Thibaud Deruelle, Veronika Kalouguina, Philipp Trein, Joël Wagner

**Affiliations:** University of Lausanne, Lausanne, Switzerland

**Keywords:** Social sciences, Economics

## Abstract

In this cross-sectional, semi-longitudinal and quasi-experimental study, our goal was to determine the effect of data storage conditions on willingness to take a genetic test. We compared individuals’ preferences regarding how they want to store health data collected from genetic tests through two survey experiments fielded in Switzerland in March 2020 and January 2022. We tested for differences whether genetic data are presented as private goods or public goods. Results confirm our initial research expectation: more control over storage increases willingness, so does framing genetic data as private good. However, they also show that the willingness to take a genetic test has noticeably increased between 2020 and 2022. Our results point toward a “pandemic effect” which would have increased willingness take a genetic test, nevertheless, more data are needed to understand this putative effect.

Willingness to share personal health data has been an increasingly relevant topic since the development of personal health records [1–3]. Beyond care, health data can harness important benefits for research, provided that individuals consent to sharing their personal data. Research shows that trust in the institutions handling the data is thus of paramount importance in explaining willingness to share [4–6]. With the digitization of health data, questions of privacy are crucial to explain willingness to share this data [7–10].

Genetic data is a particularly sensitive form of health data, and individuals’ willingness to even elect to take a test may be mitigated by their attitude toward genetic information and its use [11–14]. In this context, taking stock of individuals’ willingness to conduct such tests is of paramount importance. The literature on genetic testing has focused on individuals’ willingness to pay for genetic tests for cancer screening [15, 16], Alzheimer’s disease [17], as well as a more exhaustive analysis of health risks [18]. However, beyond the cost of tests, we need to understand how privacy concerns may be a barrier to taking a genetic test.

In this quasi-experimental, cross-sectional, and semi-longitudinal study, we analyze Swiss residents’ willingness to take a genetic test as the dependent variable. We test the expectation that privacy concerns are crucial to explain why individuals report that they are willing to take a genetic test. Specifically, we conducted two surveys on the willingness to take a genetic test, one in March 2020 and one in January 2022. We test storage conditions as independent variables. To generate a quasi-experimental approach, we randomly framed individuals into two equal-sized groups while controlling for the gender, age and language region distributions and framed genetic data as public good for one group and private good for the other (see Supplementary Material for further information).

Our main research expectations were that more control over storage increases willingness to take a genetic test, so does framing genetic data as private good, which our study confirmed [19]. However, to our surprise, the most interesting finding was that willingness to take a genetic test had noticeably increased in the period between both surveys. In the 22 months of Covid-19 pandemic separating the two surveys, individuals’ willingness to take a genetic test has increased. This finding points toward a “pandemic effect” on the willingness to take a genetic test. More generally, this paper underlines that individuals’ control over their genetic data is important and should be included in the design of biobanks [20, 21]. Below we describe the two surveys, especially vis-a‘-vis their sample and compare levels of agreement between results. We then discuss possible causes for this “pandemic effect” and reflect on future avenues for research.

## Survey data

In March 2020, we tested individuals’ willingness to take a genetic test in a field survey in Switzerland sample (*N* = 1000). Our research builds on prior studies which emphasize that individuals cherish the protection of their personal health data [6, 9, 10], especially genetic data [11, 13, 14] because it is an infringement into physical privacy [19]. We thus tested whether different forms of storage to personal health data from genetic tests, would have different effects on citizens’ willingness to take those tests.

In the survey, after briefly explaining what is meant by genetic tests, we randomly framed individuals into two equal-sized groups while controlling for the gender, age and language region distributions. One group (common good framing—CG) received the following framing: “Some consider that personal health data is a common good and should be used to improve public health.” Then we asked them how likely it would be that they take a genetic test, if their data were stored by public authorities, such as a biobank. The other group (private good framing—PG) was provided with a different framing: “Some consider that personal data are private and should be used exclusively to improve the health of the individuals to whom they belong.” After that, we asked respondents if they were willing to conduct a genetic test, if they were to store their health data themselves, for example in a”datasafe” or secure server. In January 2022, we conducted a follow-up survey in Switzerland (*N* = 1047) in which we applied the same framing and asked the same questions. Both samples were representative of the Swiss population: men and women are distributed equally, the participants, aged between 25 and 65 years, are evenly distributed into four age groups, and the same quotas for the German and French language regions are used (see Supplementary Material for further information).

## Willingness to take a genetic test before and after COVID-19

Findings show differences between the survey conducted in 2020 and the one from 2022. In both surveys, the results of our analyses show that if data is exclusively under their control, individuals are more likely to take a genetic test. However, as demonstrated in Table 1, willingness to take a genetic test is higher in 2022 by more than 20 percentage points (p.p.) for CG framing and more than 13 p.p. for PG framing for the full sample. We conducted a t-test to compare the two samples (2020 and 2022) and the difference is statistically significant.

**Table 1:** Willingness to take a genetic test before and after the COVID-19 pandemic.

	2020	2022	Difference	Significance
Full sample				
Common good	39.20%	59.35%	20.15 p.p.	***
*N* (Observations)	500	524		
Private good	51.40%	64.63%	13.23 p.p.	***
*N* (Observations	500	523		
Panel sample				
Common good	45.54%	54.55%	9.01 p.p.	*
*N* (Observations)	224	220		
Private good	47.52%	62.62%	15.1 p.p.	**
*N* (Observations)	202	206		
Changes in framing				
Same framing	47.6%	60%	12.4 p.p.	***
*N* (Observations)	220			
Framing CG -> PG	43.6%	59.1%	15.5 p.p.	***
*N* (Observations)	110			
Framing PG -> CG	47.2%	54.7%	7.5 p.p.	
*N* (Observations)	106			

Significance levels are *p < 0.05; **p < 0.01; ***p < 0.001.

Among the 1046 respondents to the 2022 survey, 426 of them were also respondents in the 2020 survey, but did not necessarily receive the same framing: we thus were able to establish a panel sample (*N* = 856). Table 1 shows that the increase in absolute change is about 9 p.p. for individuals who received the CG framing and about 15 p.p. for those who received the PG framing. We conducted a *t*-test and the difference between 2020 and 2022 is statistically significant although not as much as for the full sample (*p* = 0.0289 for CG framing and *p* = 0.0011 for PG framing). Finally, we also compare those who received the same framing in 2020 and 2022 and we observe an increase in willingness to conduct genetic testing of 12.4 p.p. Nevertheless, amongst those who changed from a CG to a PG framing, the willingness to test increased even more (15.5 p.p.), whereas the respondents that changed from a PG to a CG framing augmented their willingness only by 7.5. p.p.

## “Pandemic effect” and the acceptability of genetic testing

Results confirm our initial research expectation: more control over data storage increases willingness to test. Our findings contribute to the literature by showing that defining health data as private good regarding which, individuals maintain a control over storage is most likely to incite individuals to take genetic tests. This finding is coherent with a previous study [22], which shows that the Swiss population is willing to share their anonymised health data, although substantial concerns regarding data protection and security have been raised.

However, they also show that the willingness to perform genetic tests has noticeably increased between 2020 and 2022. Differences in framing fail to coherently explain this phenomenon. Indeed, we observe a larger increase in willingness for CG framing than for the PG framing, when comparing full samples. Nevertheless, the contrary is true if we look at the panel sample only. The timing at which the two surveys were conducted points toward a “pandemic effect” through which the Covid-19 crisis would have increased support toward genetic testing.

The motifs for this change in attitude toward testing might be multifaceted and their analysis goes beyond the scope of this paper. At this point, we can only speculate about them and how they can inform our research agenda. One motivation could be that between the two surveys, the Covid-19 pandemic might have “normalized” genetic tests in the sense, that it increased individuals’ openness to health-related testing. Another motivation could come from an increased legitimacy of genetic testing. Notably, testing for Covid-19 (voluntary tests as well as testing imposed by government) might have increased trust in testing for other diseases early on or incited individuals to conduct check-ups. Finally, another possible motivation could be increased health-consciousness. Put differently, the pandemic might have put maintaining good health higher on the agenda of individuals.

This study has limitations. More data is needed to establish the causal relation of this claim: our study did not aim to identify the cause of such changes and our explanation of those results remains speculative. The representativeness of the panel-elements in our sample is limited, as females, older people and French speaking cantons are overrepresented compared to the overall sample. Further studies could rule out sample limitations, chance, and other explanatory variables, such as trust, which were not included in this analysis. In addition, future research could dig deeper into the difference between where data is stored (private or public place) and access (control or limited control) by individuals.

Nevertheless, this study can inform policies which aim at developing prevention through genetic testing, for instance related to cancer screening. The Covid-19 pandemic might have opened a window of opportunity to advance the use of genetic data, whereas for individual care or to contribute to research. Our study shows that, this will depend on how the very concept of health data is framed to the public.

## Supplementary information

Supplementary material

## Data Availability

The datasets generated and analyzed during the current study are available in Harvard’s dataverse repository at 10.7910/DVN/CMQVCA.
