# Supplementary material for: Is there a “pandemic effect” on individuals’ willingness to take genetic tests?
Source: Eur J Hum Genet. 2022 Nov 10;31(3):360–2. doi: 10.1038/s41431-022-01223-6 (PMC9646467; doi:10.1038/s41431-022-01223-6)
Supplement: Supplementary file 1 — Supplementary material [file 41431_2022_1223_MOESM1_ESM.pdf]

Is there a "pandemic effect" on individuals' willingness to take genetic tests
Supplementary material

Thibaud Deruelle, Philipp Trein, Veronika Kalouguina, Joël Wagner*

September 12, 2022

The data is available on Dataverse
https://dataverse.harvard.edu/dataset.xhtml?persistentId=doi:10.7910/DVN/CMQVCA

1 Survey questions and framing

Definition of tests

In the 2020 survey, we defined genetic tests as: "Do-it-yourself blood or genetic tests" to be sent to a laboratory that allow to determine potential food intolerance, to suggest an optimal exercise plan or to assess the risk of certain hereditary diseases (such as cancer for instance).

In the 2022 survey, respondents were presented with similar information to our 2020 survey. However, for the 2022 survey, we used the terms "blood and genetic tests", defined as blood or saliva tests to send to the lab in order to determine possible diseases (for instance cancer). Those terms were consistent with the questions asked in 2020 which used the phrase "blood or genetic tests".

We included the term "blood" in order to prevent our respondents from assuming that genetic test would be a simple saliva test (as those performed for ancestry analyses which were mentioned in earlier questions of our survey). In doing so, we wanted our respondents to cease possible "costs" associated with conducting a genetic test, including aspects relating to convenience. This term is therefore reflected in the questions we used.

Framing and questions

For both the 2020 and the 2022 surveys, after presenting the information on genetic tests, we randomly framed individuals into two equal-sized groups (while controlling for gender, age and language region distributions).

*Thibaud Deruelle, corresponding author (thibaud.deruelle@unil.ch). This work was supported by the Swiss National Science Foundation (grant no. CRSII5_180350) which is gratefully acknowledged.

One group **common good framing, CG thereafter** received the following information: "Some people say that personal data are a common good and should be shared and used to increase knowledge on public health". We then asked them: "If this data was to be stored by a public institution, for instance in a state data bank would you use / continue using blood or genetic tests?"

The other group **private good framing, PG thereafter** was provided with the information that "Some people say that personal data are private and should be shared and used only with the agreement of each individual". Then this group was asked: "If you had to store this data yourself, for instance in a "datasafe" or secure server, how likely would you use / continue using blood or genetic tests?"

For all questions, we used a 4-point Likert-scale. Answer options were: *1. Not likely 2. Unlikely 3. Likely 4. Very likely.* For the use of the data in the paper, we coded the variables into binary measures. Those who responded that it is not likely or unlikely that they take the test were coded as "0" whereas those who answered that it is likely or very likely they take a test were coded as "1".

Variables

The dataset we publicized on the dataverse contains the following variables:

- gender: 1 "women", 2 "man"
- age: Four age classes: 1 "25-34", 2 "35-44", 3 "45-54", 4 "55-65"
- region: 1 "German-speaking", 2 "French/Italian-speaking"
- cg_gen": "Common good 4 scale". This variable measures willingness of those who were exposed the CG framing on a four item Likert-scale.
- pg_gen": "Private good 4 scale". This variable measures willingness of those who were exposed the PG framing on a four item Likert-scale.
- cg_gen_bin": "Common good binary". This variable measures willingness of those who were exposed the CG framing as a binary variable.
- pg_gen_bin": "Private good binary“. This variable measures willingness of those who were exposed the PG framing as a binary variable.

2 Representativity

Descriptive statistics table

	Samples					
	2020		2022		Panel sample	
	N	(Share)	N	(Share)	N	(Share)
**Gender**						
Male	500	(50.0)	523	(50.0)	390	(45.77)
Female	500	(50.0)	523	(50.0)	462	(54.23)
**Age**						
25 – 34	250	(25.0)	238	(22.8)	160	(18.78)
35 – 44	250	(25.0)	262	(25.0)	203	(23.83)
45 – 54	250	(25.0)	306	(29.3)	230	(27.0)
55 – 65	250	(25.0)	240	(22.9)	259	(30.4)
**Language region**						
French	330	(33.0)	345	(33.0)	332	(39.09)
German	670	(67.0)	701	(67.0)	519	(60.92)
Sample N	1 000	(100.0)	1 046	(100.0)	852	(100.0)

Panel sample

About half of our respondents participated both in the 2020 and the 2022 survey. Our panel sample is thus smaller ($N = 852$) as well as less representative of the Swiss population than the 2020 and the 2022 samples.

In terms of sex, females ($N = 462$) are over represented compared to males ($N = 390$), while the 2020 and the 2022 samples are both equally divided between sex.

In terms of age group, the 55 to 65 years old cohort is over represented at 30.4% ($N = 259$), and the 25 to 34 years old cohort is under-represented at 18.78% ($N = 160$) , as the four age cohorts are equally represented in the 2020 sample and roughly equally represented in the 2022 sample.

Finally in terms of linguistic regions, the French speaking region is slightly over represented in the panel sample, at 39% ($N = 332$) instead of 33% as in both the 2020 and 2022 samples.
